# Supplementary figures and images for: Adaptive evolution among cytoplasmic piRNA proteins leads to decreased genomic auto-immunity
Source: PLoS Genet. 2020 Jun 11;16(6):e1008861. doi: 10.1371/journal.pgen.1008861 (PMC7310878; doi:10.1371/journal.pgen.1008861)

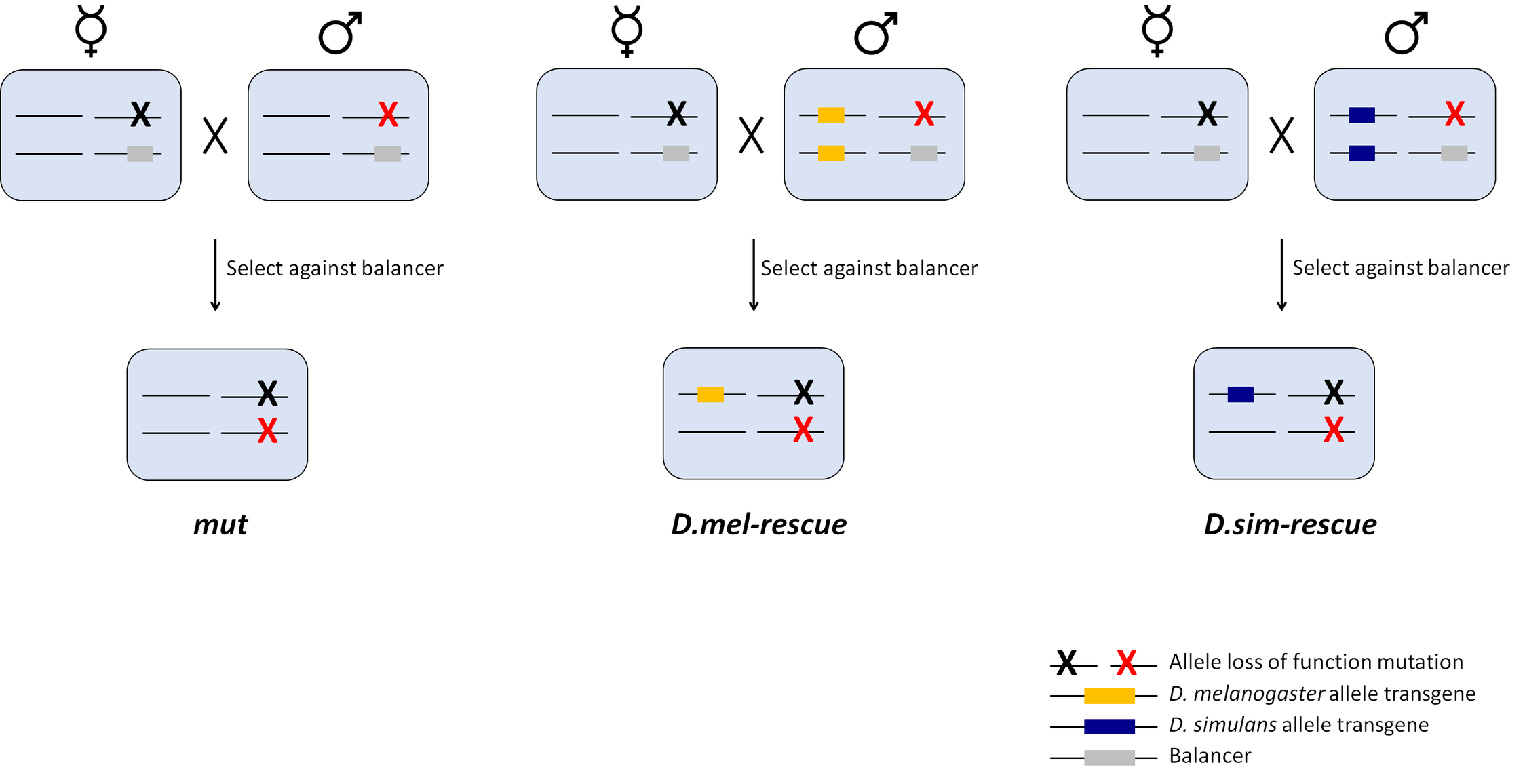

Supplement: S1 Fig — (TIF) [file pgen.1008861.s001.tif]

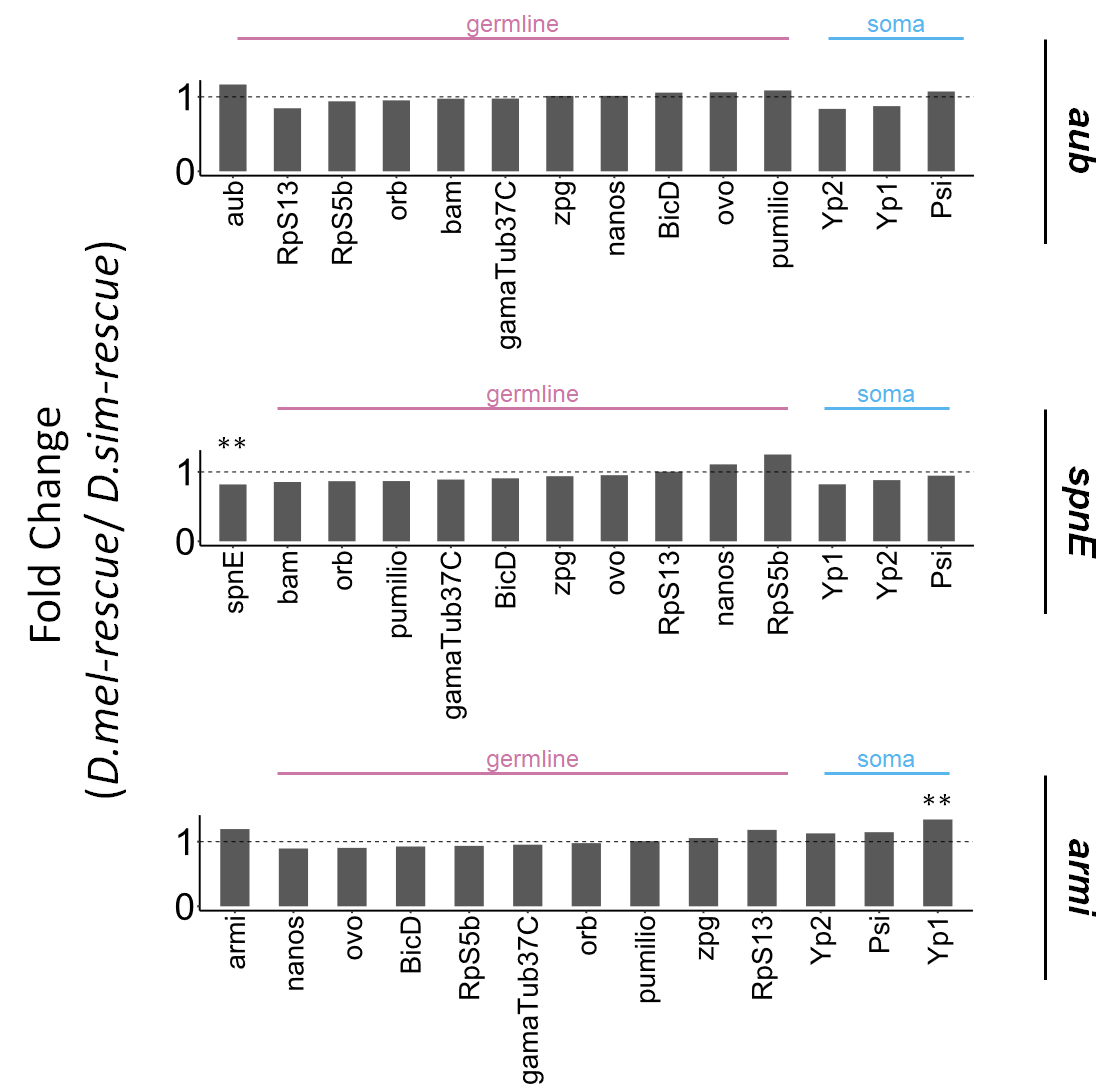

Supplement: S2 Fig — Fold-change of expression level of aub, spn-E, armi, germline-specific genes and soma-specific genes between D. melanogaster transgenic rescue and D. simulans transgenic rescue are shown. Fold-change values are based on one biological replicate for aub and three biological replicates for spn-E and armi, and were obtained from a DESeq analysis for aub and a DESeq2 analysis for spn-E and armi. ** denotes p ≤ 0.01. NS if not labeled. (TIF) [file pgen.1008861.s002.tif]

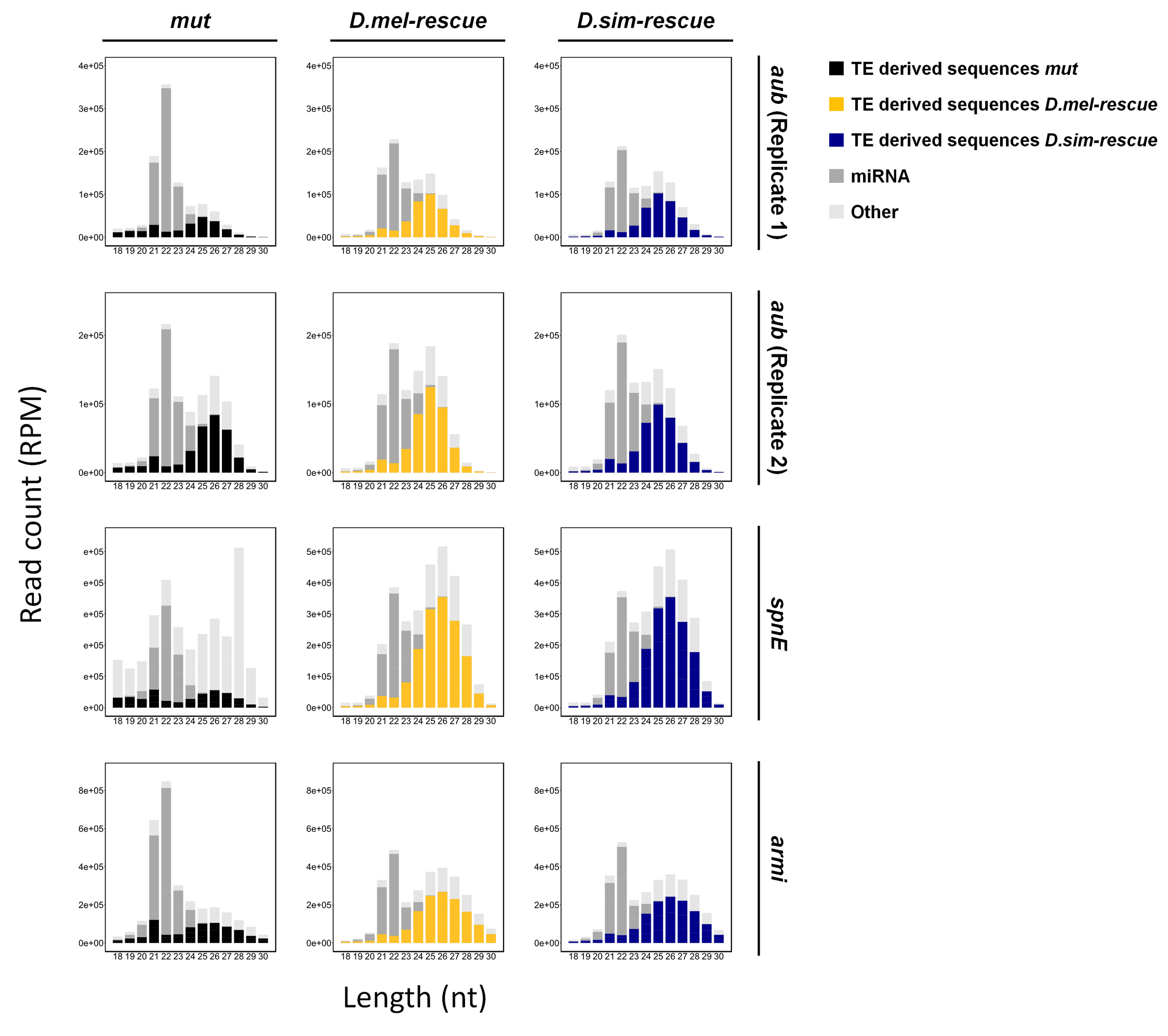

Supplement: S3 Fig — (TIF) [file pgen.1008861.s003.tif]

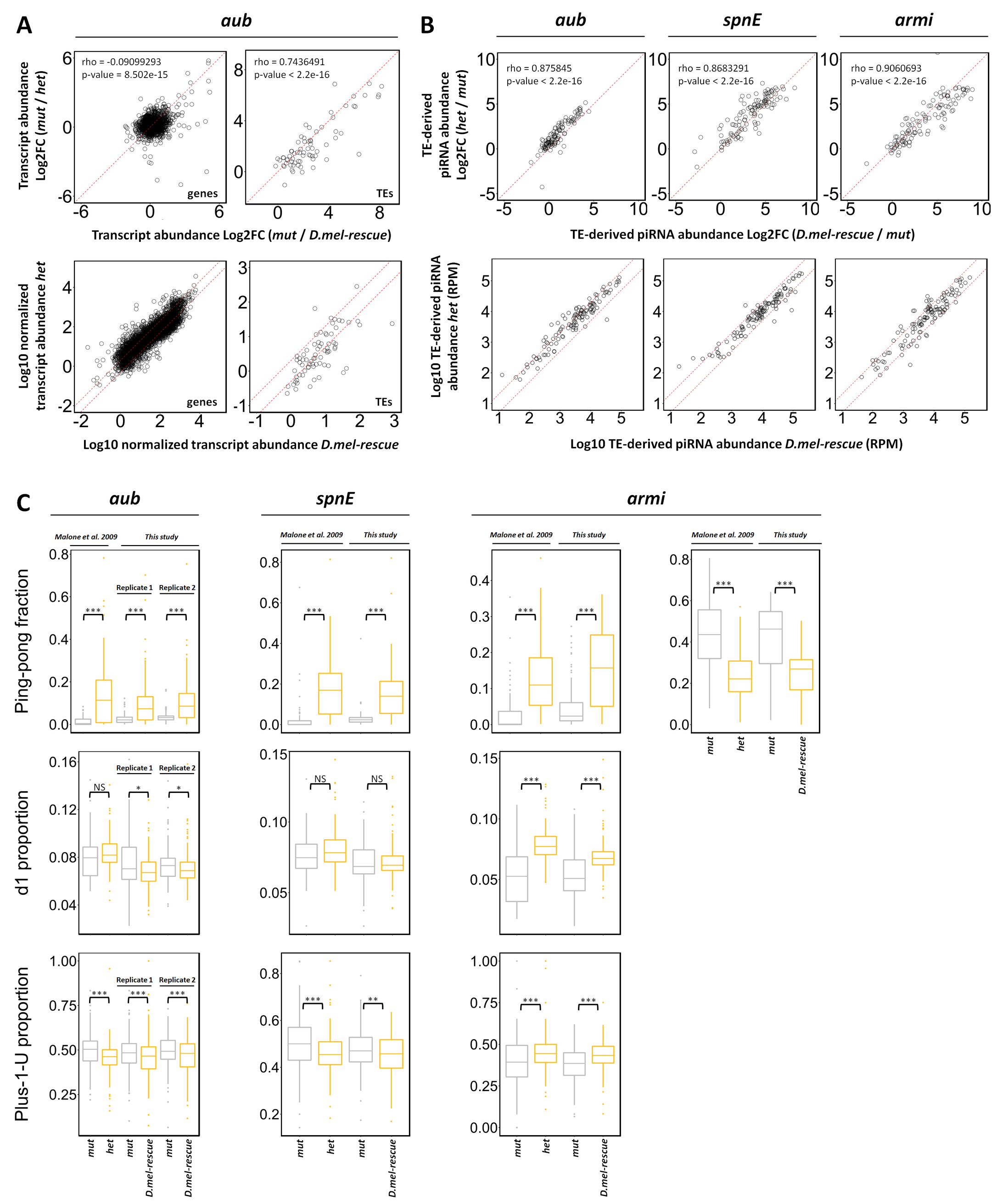

Supplement: S4 Fig — Drosophila melanogaster transgenes and heterozygotes are compared to trans-heterozygous mutants with respect to transcript abundance (A), TE-derived piRNA abundance (B), ping-pong and phasing biogenesis (C). RNA-seq data comparing aub heterozygotes and trans-heterozygous mutants is from [70]. Transcript abundance was normalized to the total number of mapped reads of that library. The small RNA-seq data comparing heterozygotes and mutants for aub, spn-E and armi are from [71]. TE-derived piRNA abundance was normalized to the total number of sequenced miRNAs in the same library. Statistical significance was assessed by the Wilcoxon signed-rank test. For aub, two biological replicates of each genotype generated at different times are shown separately. For spn-E and armi, averages of three biological replicates of each genotype generated at the same time are shown. NS denotes p > 0.05. *, **, and *** denote p ≤ 0.05, p ≤ 0.01, p ≤ 0.001, respectively. (TIF) [file pgen.1008861.s004.tif]

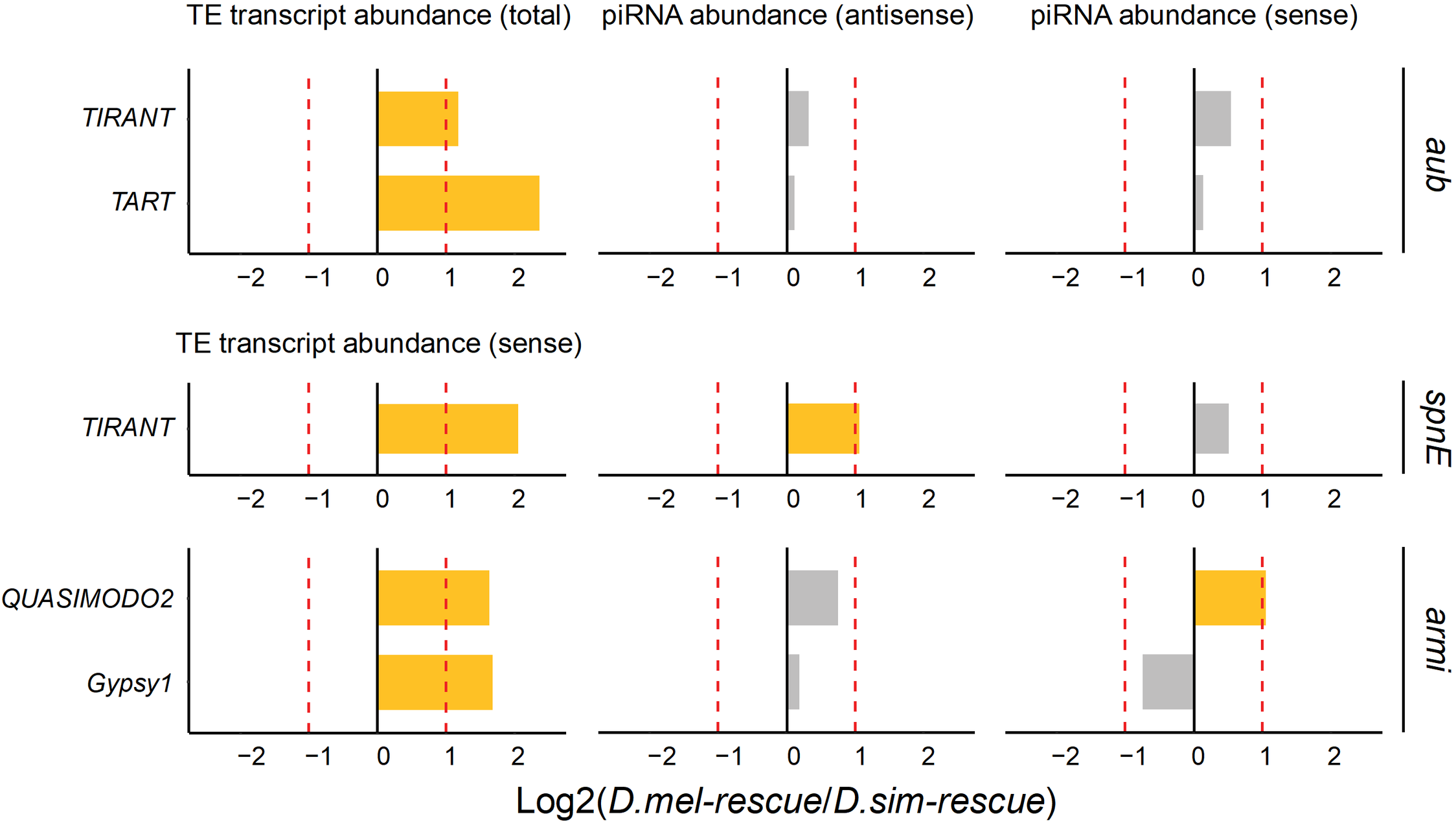

Supplement: S5 Fig — Log2 fold-change TE transcript abundance and TE-derived sense/antisense piRNA abundance between two transgenic rescues for the TE families whose TE transcript abundance is substantively different (> 2 fold) between two rescues from Fig 2A. Red dashed lines indicate the 2 fold-change threshold. (TIF) [file pgen.1008861.s005.tif]

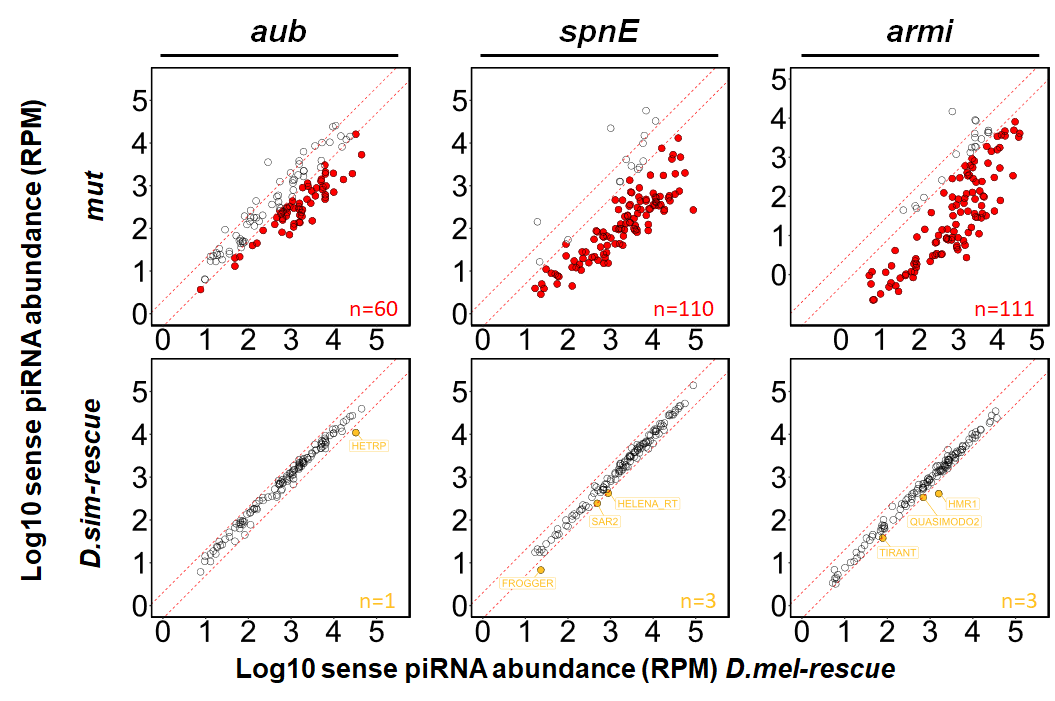

Supplement: S6 Fig — (TIF) [file pgen.1008861.s006.tif]

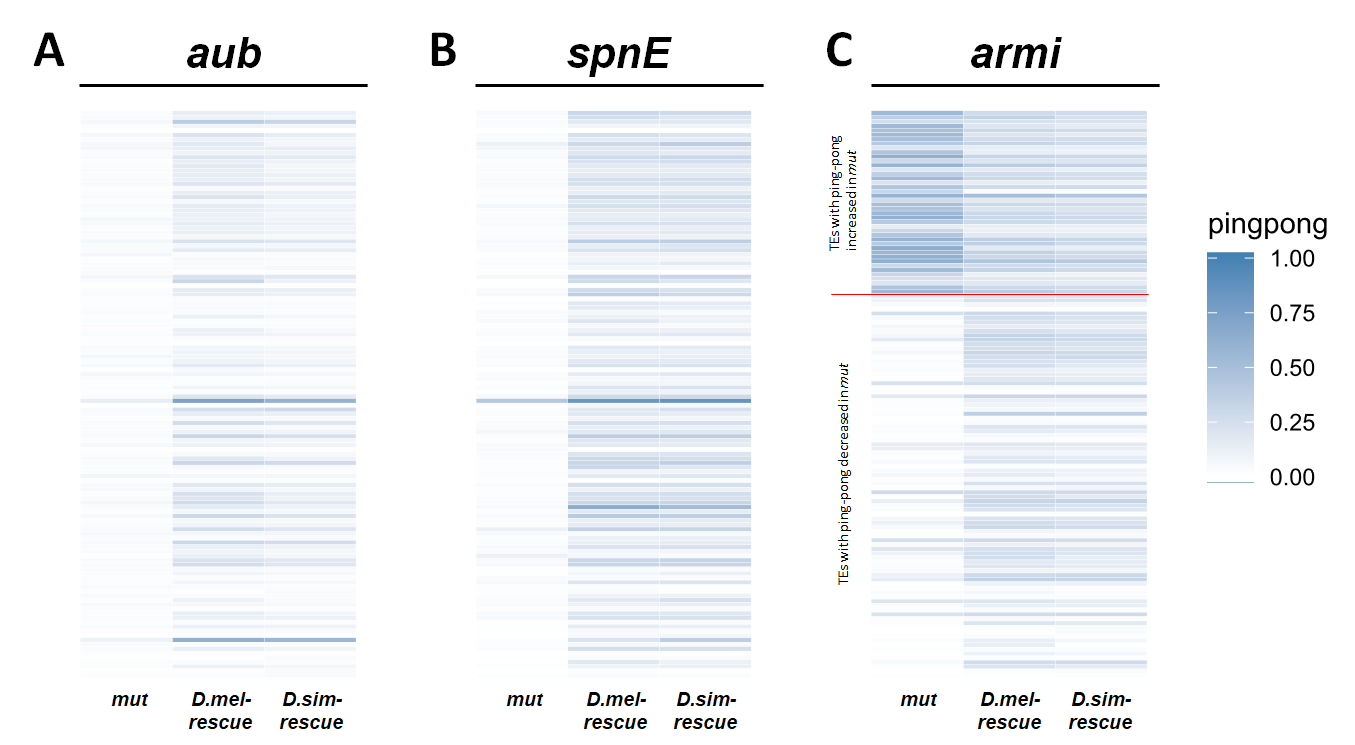

Supplement: S7 Fig — (A) aub, (B) spn-E and (C) armi. Among (C), 88 and 42 TE families whose ping-pong fractions are decreased (below red line) or increased (above red line), respectively, in armi mutant as compared to those in D. melanogaster transgenic rescue are shown. (TIF) [file pgen.1008861.s007.tif]

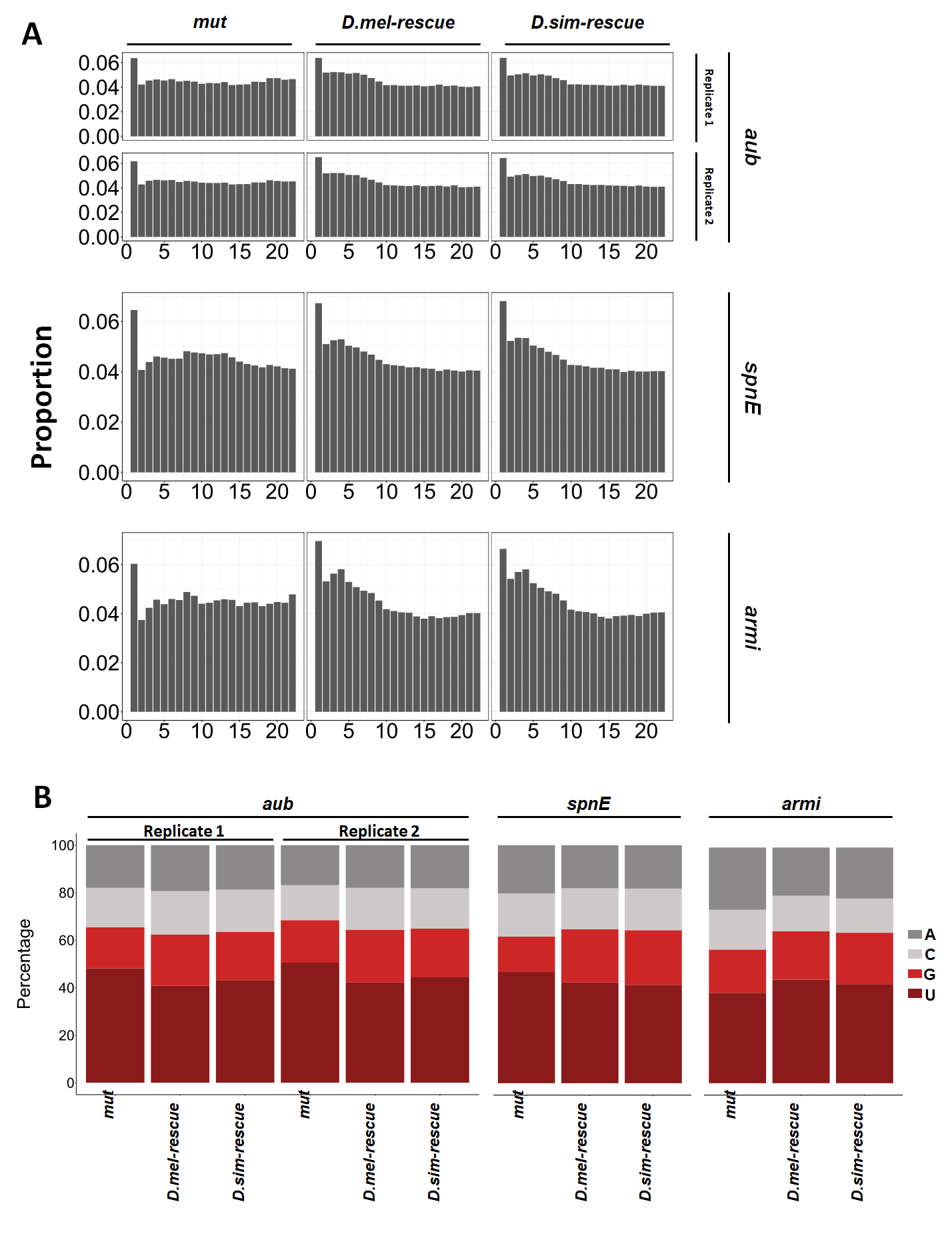

Supplement: S8 Fig — Observed peaks of 1nt distance (A) and +1-U bias (B) among each genotype for each protein studied. (TIF) [file pgen.1008861.s008.tif]

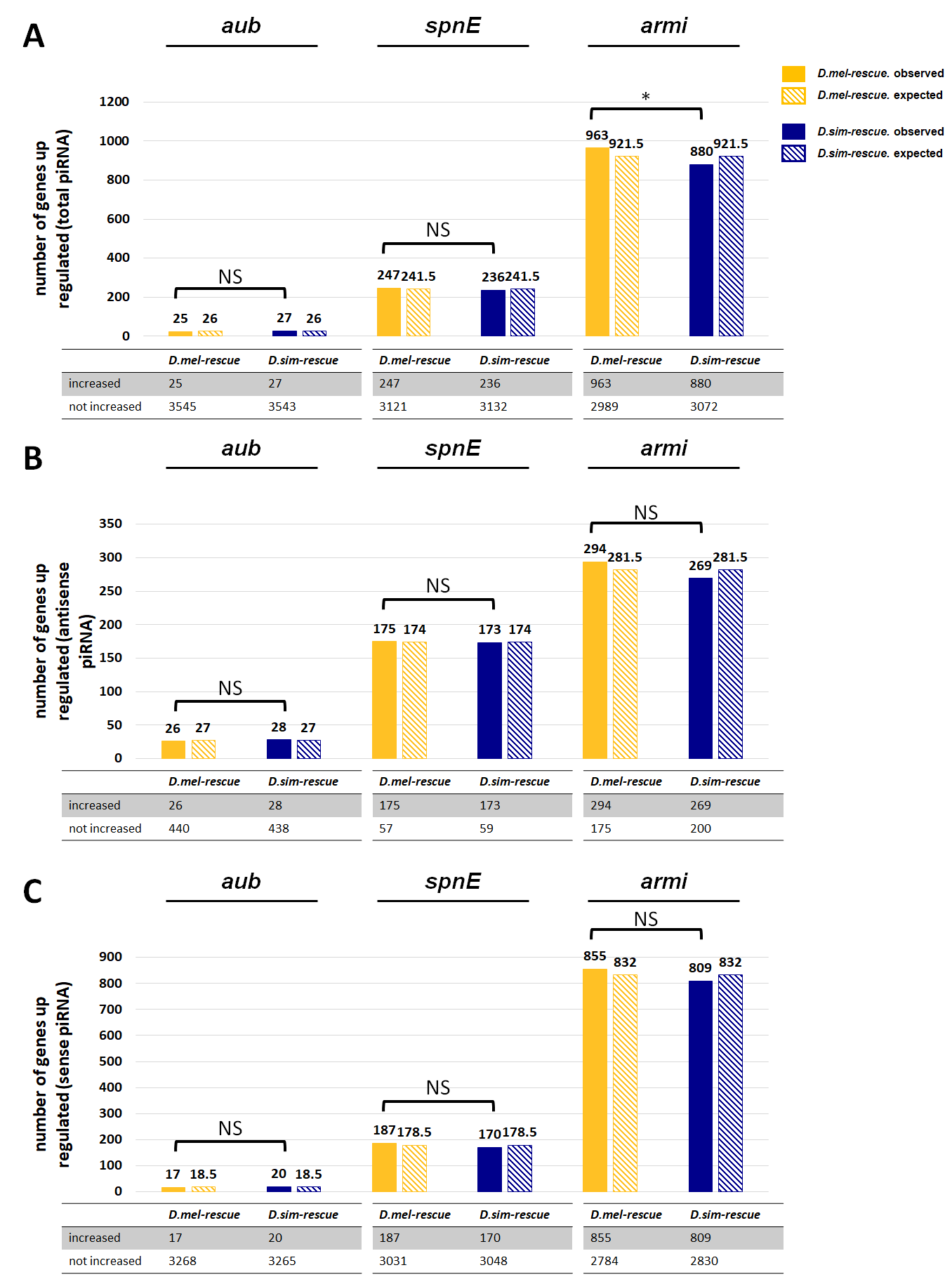

Supplement: S9 Fig — The number of genes whose corresponding total (A) / anti-sense (B) / sense (C) piRNA abundance is increased (>1.5 fold) in the presence of each transgene as compared to the mutant. Contingency tables are shown below. Log2 fold-change values were based on two biological replicates for aub and three biological replicates for spn-E and armi, and were obtained from a DESeq2 analysis (adjusted p < 0.05). Statistical significance was assessed by the Pearson's Chi-squared test. NS denotes p > 0.05. * denotes p ≤ 0.05. (TIF) [file pgen.1008861.s009.tif]
