# Supplementary material for: Adaptive evolution among cytoplasmic piRNA proteins leads to decreased genomic auto-immunity
Source: PLoS Genet. 2020 Jun 11;16(6):e1008861. doi: 10.1371/journal.pgen.1008861 (PMC7310878; doi:10.1371/journal.pgen.1008861)
Supplement: Attachment — Submitted filename: Wang_et_al_revision_review20200212.pdf [file pgen.1008861.s022.pdf]

Comments of the Editor:

1) Please clarify whether the data in Figure S3 is total piRNAs, or restricted to those that map to TEs.

They are TE-derived piRNAs. Added.

2) Typo in table associated with figure 4A; ‘reguated’

Changed.

3) Consider replacing Figure 4 panels D and E with analogs of Figure 2B. If not, please improve the figure legend.

Our purpose in Panel 4D is to ask if sense and antisense piRNAs corresponding to downregulated genes are systematically increased in the D. simulans *rescue. We agree panels 4D and E are confusing, however we do not think that a scatterplot is the best way to visualize these results. The sign-test we perform (a non-parametric test asking if log2FC values systematically differ from zero) is best visualized by comparing observed log2FC values to zero, or observed proportions log2FC >< 0 to 50/50. We therefore generated a bar graph that clearly conveys the proportion of genes with sim bias (log2FC < 0) and mel bias (log2FC > 0) as well as the sample size (Figure 4E).*

4) Consider revising Table S9 so that the reader can more easily see the increase in the D. simulans sense piRNAs.

We revised table S9 to provide the log2FC and p-values for mel vs sim comparisons of mRNA, sense piRNA, and antisense piRNA.

5) Consider the analysis of the already available aub-iCLIP data suggested by the reviewer; I agree that, if the data are appropriate, this analysis would strengthen the evidence for promiscuous binding of mRNAs.

We also felt this was an interesting analysis that would strengthen the evidence of binding of host mRNAs. We therefore compared the 132 genes that are down-regulated by Aub in our study with the 634 genes in the Aub-iCLIP study by Barckmann et al. 2015, and determined that downregulated genes are significantly enriched in the iCLIP data. We included this observation in the revised auto-immunity section.

6) Please take care to italicise ‘D. melanogaster’ and ‘D. simulans’.

Done.

7) Please note the other minor issues raised by reviewers 2 and 4.

Done, see below.

Comments of Reviewer 2:

1) Figure S3B – 2nd row 2nd column the top of the axis is cut off.

Changed.

Comments of Reviewer 3:

1) In figure S3A it is not clear from the legend whether the plot is for piRNA abundance in the library or piRNA abundance to TE transcripts (similar to figure 2B). Also, can the authors include a panel in figure S3 similar to figure 2B just comparing piRNA abundance between Dmel-rescue and hets with a 2-fold line.

Done. In figure S3AB (previously S3A), we modified the figure and legend to clearly state that the piRNAs are TE-derived. We also added another panel comparing the piRNA abundance between Dmel-rescue and hets with a 2-fold line, as the reviewer requested.

2) Similar to small RNA data comparisons for het vs. mel-rescue (Malone et al. 2009), RNA-seq datasets for het or WT vs mutant (aub, armi and spn-E) are available. As a control, it would be helpful to show a comparison between het vs mel-rescue for RNA-seq data. Or, if the authors can show from previous studies comparing mut vs. het (aub, armi and spn-E) that the overlap in genes up/downregulated is the nearly the same as mel-rescue vs. mutant comparison (and that more genes tend to be downregulated than upregulated), this will be additional support that mel-rescue indeed behaves as het (WT) for RNA-seq data.

The only RNA-seq data we are able to locate is from aub (Teixiera et al*. 2017). With the caveat that it is not strictly appropriate to compare RNA-seq data sets that were generated in different labs and via different library prep methods, we do observe a strong correlation in gene expression and TE expression between libraries. These analyses are in Figure S3A.*

3) In the revised manuscript, the authors observe off-target effects are not due to piRNAs (lack of evidence for anti-sense piRNAs to mRNAs), but instead observed an increase in sense piRNAs, which is consistent with a model of promiscuous binding of piRNA pathway proteins to process mRNAs into piRNAs. Figs. 4 D and E are not very informative. Are the blue and grey dotted lines expected vs. observed? This is not mentioned in the figure legend. Wouldn't it be more informative if the authors show this result similar to Fig. 2B? This would be a nice locus-by-locus comparison of sense piRNA levels like the authors do for TE anti-sense piRNAs in Fig. 2B.

See our response to editorial comment 3.

4) Also, in table S9 if the log2FC values are interpreted as 2-fold changes for sense piRNA production (similar to what the authors do for TEs), what fraction of genes in aub and armi have 2-fold more sense piRNAs in D.sim rescue compared to D.mel rescue? If the comparisons are from DEseq2, provide adjusted P for every gene where replicates are available, similar to table S3. If promiscuous binding of piRNA effector proteins generates more sense piRNAs from mRNAs on a model that the authors propose, wouldn't there be significantly more sense piRNAs (1.5 fold or more) for every individual downregulated locus in D.sim rescue compared to D.mel rescue (similar to anti-sense piRNAs for TEs; fig 2B)? From table S9, I am not sure if the data strongly support this model.

In fact, we did not consider a log2FC threshold or a P-value in the analysis for Figure 4D (now 4E), since we were assessing a systematic change in piRNA abundance across a class of genes, rather than an individually significant change in piRNA production. Rather, log2FC and P-value cutoffs were applied only in Figure 4A-C, where we identified genes significantly downregulated by either transgene when compared to the mutant. These genes tend to exhibit lower expression levels in the presence of the D. simulans *allele, however the changes are largely not individually significant. Similarly, sense piRNAs from these genes tend to exhibit higher expression levels in the presence of the* D. simulans *alleles, however, these changes are also largely not individually significant. This is fully consistent with promiscuous binding by D. simulans alleles leading to modest reductions in gene expression and corresponding increases in sense piRNA production.*

In the revised manuscript we have edited the genomic autoimmunity section considerably to better emphasize the subtlety of the off target effects. We have also generated a revised table s9 that shows log2FC changes in gene expression and sense and antisense piRNA production between transgenes for the downregulated genes.

5) One other possibility is that binding of piRNA effector proteins to mRNAs may promote mRNA degradation (see Barckmann et al. 2015). As the suggested selection for

genomic autoimmunity is a critical point in this manuscript, the authors can examine if the downregulated genes in their comparison are bound by aub in the aub iCLIP data in Dmel from Barckmann et al. 2015 to support this model (the authors show large fraction of downregulated genes are shared between Dmel and Dsim; Fig 4C). The data is from 0-2hr embryos, but mRNA population in the ovary is represented in the maternally deposited pool, and either this or analysis of similar CLIP data may lend strong support for the model that the authors propose in this manuscript.

We had not considered the possibility that piRNA protein binding to mRNAs could also promote degradation; we thank the reviewer for pointing this out. We mention degradation as an alternative mechanism for piRNA proteins reducing host gene expression in the genomic autoimmunity section.

We thank the reviewers also for their suggestions of the iCLIP data set, which we incorporated into the revised manuscript (see our response to editor's comments).

6) The authors provide supplementary data tables for sRNA and RNA-seq data in the revised manuscript. For table S9, if the comparisons are from DEseq2, provide adjusted P similar to table S3 and S8.

   Done.

Comments of Reviewer 4: This article is greatly improved for clarity and it seems the major concern this reviewer had was resolved by identifying and fixing an artifact in the previous analysis.

My only suggestion is for greater clarity in figures 4D and 4E. The represented probability distributions are estimated from the data, but they should overlay the actual data on these plots. In addition, the following values on the plots should be defined in the figure legend:

1) What is 's'? It looks like the number for LogFC2 >0, but I would recommend deleting that because it is both redundant and confusing.

See our response to editorial comment 3.

2) Please clarify that the dotted grey line is the zero point and the dotted blue line is the mean(?)/median(?)..

See our response to editorial comment 3.

3) Label the three panels in D and E both with aub, spnE and armi? I assume that is the order, but it is not 100% clear.

See our response to editorial comment 3.
