# Supplementary material for: Adaptive evolution among cytoplasmic piRNA proteins leads to decreased genomic auto-immunity
Source: PLoS Genet. 2020 Jun 11;16(6):e1008861. doi: 10.1371/journal.pgen.1008861 (PMC7310878; doi:10.1371/journal.pgen.1008861)
Supplement: Attachment — Submitted filename: Wang_et_al_revision_review20200420.pdf [file pgen.1008861.s023.pdf]

Abstract Line 17: stray 't'

Deleted.

p. 8 "Samples sizes" => Sample sizes

Changed.

p. 8 Line 8: " Because D. simulans transgenes do not exhibit significantly reduced expression when compared to D.melanogaster (S2 Fig)": Unless I've misread Figures S2, it shows 'significantly reduced expression' for the D simulans spnE transgene. Please clarify.

In fact the D. simulans spnE transgene exhibits a 22% increase in expression when compared to D. melanogaster, which is consistent with our suggestion that reduced fertility in the presence of D. simulans alleles does not reflect reduced expression. We have edited the text to highlight this result.

p. 9 "This surprising bias towards enhanced negative regulation by D. simulans is not consistent with the random segregation of TE copies during backcrossing."; please clarify what this means.

When backcrossing transgenes into heterozygous mutant backgrounds, it is possible that some small fraction of TE insertions will differ between D. melanogaster and D. simulans transgenic stocks, corresponding to insertion polymorphisms between the injected and mutant stock that differentially segregate. These presence/absence differences for individual TE insertions could potentially lead to expression differences between the two transgenes if the TE copy number is low or the insertion in question is highly expressed. However, such background effects would be random with respect to the identity of the transgene, with some families showing higher expression in the D. simulans transgenic rescue and others showing higher expression in the D. melanogaster transgenic rescue. That differs from what we observe, which is that differentially expressed TEs always exhibit reduced expression with the D. simulans allele. This suggests a systematic, biologically-based regulatory difference between transgenes.

We clarified our language in this section to better articulate this observation.

p. 9, Lines 3 & 15, "5 out of 5 TE families": I think there are only 4 families, as Tirant appears twice.

Tirant does appear twice. However because the backcrossing of each transgene is an independent event, enhanced negative regulation of tirant by D. simulans spn-E and aub should be considered independent observations. We have clarified our language to make this more clear.

Fig 2. Why is total TE transcript level shown for aub, rather than only sense as for the other two proteins? Non-stranded libraries? If so, that's fine, but please clarify. Why are sense TE piRNAs never shown?

RNA sequencing for these three proteins were performed at different times and with different sequencing methods. For aub, we used unstranded mRNA sequencing, for spn-E and armi, we used stranded total RNA sequencing. This was stated in the methods, however, we edited the Fig 2 legend to clarify.

We focus our discussion on antisense piRNAs as they are responsible for targeting TE regulation. However, we added the sense piRNA results as a supplementary figure (S5 Fig), and added a brief discussion of these results.

Fig. S4-- What do the rectangles represent- is it the range between the two? Also, it would be helpful to remind the reader of which two transgenes from figure 2A are being compared/

This is a horizontal bar graph and the rectangles are bars, which represent the log2 fold-changes of the piRNA/TE transcript abundance between the two transgenic rescues. This is shown on the X-axis "Log2(D.mel-rescue/D.sim-rescue)".

We can see how this figure was confusing because there was not a vertical line indicating log2FC = 0. We have added this line to the panels, as well as included a representation of sense piRNAs.

Please read over the supplementary figure legends, and make sure they are complete.

Done.

Please also consider mentioning the (admittedly modest) differences in piRNA biogenesis in the abstract, and consider clarifying that these do not result in global piRNA differences at the beginning of this section, as otherwise the reader is left wondering about this point.

Done.

In addition we ask that you:
Provide a striking Image with a corresponding caption to accompany your manuscript if one is available (either a new image or an existing one from within your manuscript). If this image is judged to be suitable, it may be featured on our website. Images should ideally be high resolution, eye-catching, single panel square images. For examples, please browse our archive. If your image is from someone other than yourself, please ensure that the artist has read and agreed to the terms and conditions of the Creative Commons Attribution License. Note: we cannot publish copyrighted images.
