# Supplementary material for: Adaptive evolution among cytoplasmic piRNA proteins leads to decreased genomic auto-immunity
Source: PLoS Genet. 2020 Jun 11;16(6):e1008861. doi: 10.1371/journal.pgen.1008861 (PMC7310878; doi:10.1371/journal.pgen.1008861)
Supplement: Attachment — Submitted filename: Wang_et_al_2019_review.docx [file pgen.1008861.s019.docx]

piRNA pathway proteins show signatures of adaptive evolution in Drosophila. What drives the rapid evolution of the piRNA machinery is unclear, but one prevailing hypothesis is that the host-TE arms race may contribute to the adaptive evolution of piRNA pathway factors. Functional consequences of rapid divergence have been studied for key piRNA factors though inter-species complementation tests, including an earlier study from the authors of this paper for Aub (Kelleher et al. 2012). Similar recent studies have tested the functional consequences of rapidly evolving nuclear piRNA factors Rhino, Deadlock (Parhad et al. 2017) and Cutoff (Parhad et al. 2019, bioarxiv) in inter-species complementation and structure-function tests to examine rescue of phenotypes with D. simulans alleles in D. melanogaster mutant background. This study is an interesting addition in testing functional consequences of rapid divergence in piRNA pathway components via inter-species complementation test, expanding on their earlier test (for Aub).

In this paper, Wang et al. test the functional consequences of rapid divergence of two additional cytoplasmic piRNA pathway factors armitage (armi) and spindle-E (spn-E). armi shuttles piRNA precursors between the ping-pong site (nuage) and the outer mitochondrial membrane where biogenesis of phased piRNAs occurs (Ge et al. 2019). Spn-E is helicase and co-IP experiments show they interact with other piRNA pathway factors such as Qin, Ago3 and Aub (Andress et al. 2016) indicating its role in piRNA amplification cycle in the nuage.

1. For fertility tests, the authors show D. simulans alleles are sub-fertile (sterility is rescued), but it seems that in terms of phenotype rescue, the adaptive evolution in the cytoplasmic factors (tested here) are different from nuclear factors like Rhino, Deadlock and cutoff, where D. simulans alleles fail to rescue sterility phenotype. I am raising this to know if these piRNA factors were to be ranked based on dN/dS or other types of selection tests the authors performed, do nuclear factors experience more positive selection than the cytoplasmic ones?

I see Rhino-deadlock work mentioned in the discussion leading to figure 6, but also work on another interacting factor cutoff is now on Bioarxiv that the authors can include in the discussion. It would be informative to include details as a rationale for why the authors chose these factors for complementation tests. Do they have significant changes in their critical domains? This information would be very helpful to interpret functional consequences, as recent co-IP studies (Andress et al. 2016, Ge et al. 2019) show that these factors interact with a host of other piRNA biogenesis and non-piRNA pathway proteins.

The authors propose a model for armi suggesting its enhanced affinity to binding ago3/aub may explain the phenotype observed (reduced ping-pong, and reduction in phased piRNAs), but it seems from co-IP that spn-E also interacts with armi, ago3 and aub, and perhaps co-evolution to maintain a robust interaction complex in the cytoplasm an additional target of selection? In the absence of structure-function tests for these piRNA factors, a detailed picture of where non-synonymous changes lie (e.g. concentrated on critical domains, or spread across the entire gene) within these genes, and whether they impact interactions with other genes can be included and discussed.

2. For Figure 1, please include the number of TE families (circles) in the panel and the “n” that is changing. At 2-fold difference how many TE families exhibit a change in the armi D. simulans rescue? I noticed the top panel is 2-fold difference and the bottom one is 1.5, is there a reason for having different fold difference cutoff? If not, just use 1.5 fold difference. Also, it would be very helpful to evaluate a supplementary table with library mapping statistics for the small RNA data. In addition, as the authors observe a drop in piRNA pool in the D. simulans armi rescue, it would be helpful to see small RNA mappings to different annotations as a pie-chart (miRNAs, coding, etc.) so I can evaluate what fraction of piRNA pool is lost in these libraries (and eventually taken-up by other categories in the library). I agree with miRNA normalization for these datasets, but it would be helpful to see the mapping fraction of libraries to different annotations for all datasets as a supplementary figure.

3. For figure2, was the experiment designed to evaluate ping-pong and phasing in WT, so the authors can add a comparison of mutant, WT, mel-rescue and sim-rescue? I agree the authors were careful in integrating transgenes into matched attP sites, but if the data is the available, it would be helpful to evaluate any background effects in ping-pong and phasing between WT and mel-rescue.

4. Same as figure1, for figure 3, please include “n” changing in the figure panel, and use consistent 1.5 or 2 fold differences.

5. For the off-targets effects, it was hard for me to evaluate this result without the details from differential expression analysis. It would be helpful to evaluate if the authors provided a supplementary table with the genes that show up and down regulation. I am not entirely convinced by this result, perhaps due to lack of underlying data to evaluate the results. The authors do not observe any up regulation of genes with D. simulans and/or D. melanogaster rescue at all? For any RNA-seq analysis, I am assuming there will be indirect effects on genes that are up/down regulated, so a comparison of D. melanogaster WT and D. melanogaster rescue RNA-seq would be a good control to assess background effects, if any. Also, for the “candidates” for off-target effects, please provide examples as a supplementary figure to show complementarily, number of piRNAs detected etc. A related question is that did the authors enrich 3’ modified small RNAs for small RNA sequencing. If not, the authors can discuss based on mapping to these protein-coding genes that they are not likely degradation of mRNAs, but genuine piRNAs. Also it is not clear from figure 4 if all the down-regulated genes are targeted by piRNAs, or some of these just secondary effects in down-regulation of genes?

Minor comments:

Figure 5A, label Y-axis (no. of genes).

Figure S6, label Y-axis (no. of genes).

Supplementary data tables for the underlying small RNA and RNA-seq data, both library stats and processed downstream files would be very helpful to evaluate results.

Figure 3 and figure S5, the authors acknowledge idiosyncratic differences in expression of individual TE families and conclude majority of TEs are not differentially expressed. I agree with this result, but the yellow (dots in figure 3, and bars in figure S5) are from D. melanogaster rescue. It would be very useful to make a comparison between WT and D. melanogaster rescue so just background effects can be evaluated. This is an important control in assessing background effects, and reflects my comments made in point 3.

The authors can cite Parhad et al. (Bioarxiv; complementation test for cutoff) in the discussion.
