# Supplementary material for: Adaptive evolution among cytoplasmic piRNA proteins leads to decreased genomic auto-immunity
Source: PLoS Genet. 2020 Jun 11;16(6):e1008861. doi: 10.1371/journal.pgen.1008861 (PMC7310878; doi:10.1371/journal.pgen.1008861)
Supplement: Attachment — Submitted filename: Wang2020RtoR.docx [file pgen.1008861.s020.docx]

We thank the reviewers for the extensive comments and reply to them below.

Reviewer #1: The fast evolution of piRNA pathway genes has been widely observed, but the underlying evolutionary mechanism has yet to be elucidated. In this study, authors used interspecific (D. melanogaster vs D. simulans) complementation tests of three fast-evolving piRNA pathway genes to investigate this question. While the authors found a significant difference in piRNA biogenesis between mutants with D. melanogaster vs D. simulans transgenes, they found little difference in terms of the TE transcripts. In addition, the authors found that there are more piRNAoff-target effects on host genes in mutants with D. simulans transgene than those with D. melanogaster transgene. Authors concluded that the main driving force for the fast evolution of piRNA pathway genes might come from piRNA production, instead of abundance, and selection for minimizing the piRNA off-target effects on host genes.

This study addresses an important question and could be of wide interests. However, there are some potential technical caveats, and additional analysis/clarifications may be needed to support authors’ conclusions fully. I listed my specific comments below.

1. Potential dosage effect of Aub, Spn-E, and Armi

According to methods and Figure S1, there is only a copy of the transgene in a null mutant background, which is usually used for rescue experiment. However, many piRNA pathway proteins work in concert, and I am wondering whether there could be dosage imbalance that would influence the biogenesis of piRNAs/TE suppression.

*Wild-type alleles of piRNA effector proteins are generally considered to be haplosufficient with respect to TE regulation and piRNA production, and it is typical to compare transheterozygous mutants to heterozygotes (mutant/wild-type) in studies of piRNA effector protein function. In the revised manuscript we demonstrate that single copy transgenic rescues confer similar rescue of piRNA production and TE suppression to that of a heterozygote as compared to mutant animals (Figures S3). While we can’t rule out that phenotypic effects of two copies may differ from those of a single copy, it is clear that the transgenes are sufficient to establish piRNA mediated silencing. Furthermore, D. melanogaster and D. simulans transgenic rescues are investigated at the same dose in matched backgrounds, thus, dosage effects do not impact any observed differences between them.*

2. What is the appropriate baseline?

All the phenotypes assayed (female fertility, piRNA biogenesis, and TE transcripts) are compared to D. mel-rescue. However, it may be necessary also to show that D. mel-rescue has similar phenotypes as those of wildtype/background strains, demonstrating that the D. mel transgene indeed rescue. This issue may be especially important given issues raised in (1). It is possible that single-copy D. mel and D. sim rescues both deviate from the wildtype and this results in little difference observed between D. mel and D. sim rescues.

*We think this is mostly addressed by our demonstration that transgenic rescues behave like heterozygotes, and that piRNA pathway proteins are generally considered dominant. However we wish to reiterate that regardless of how the transgenic rescues do or do not differ from wild-type, the goal of our study is to detect interspecific differences between the transgenes. Our experiment is well designed to do so by eliminating potential background effects that could confound our results.*

3. TE families with large divergence in abundance

Although the authors did not find large global differences in TE transcripts between D. mel and D. sim rescues, there are still some families with different transcripts abundance between the two. I am wondering whether these are families that show the largest abundance difference between the two species. It could also be informative to analyze the divergence in piRNA biogenesis with respect to TE copy number difference between the two species.

*At the suggestion of this reviewer, we investigated more carefully the evolutionary history of TE families that are differentially expressed between transgenes, including evaluating the relationship between differential TE transcript and differential TE copy numbers between D. melanogaster and D. simulans, as estimated from a previously published population genomic study (Kofler et al 2015). While we observed no systematic effect, we did observe that the Tirant element, which is more robustly silenced by the D. simulans alleles of both aub and spnE, has well characterized differences in dynamics between the two species. We discuss these dynamics in light of our observations in the results and discussion of the revised manuscript.*

4. Off-target effects of piRNAs on host genes

What authors observed (fewer protein-coding genes with increased piRNA abundance in D. sim rescue than D. mel rescue) seems to contradict with their findings and interpretations (D. sim rescue leads to more off-target). More explanations/further investigations may be needed. Also, the authors suggest that this phenomenon is similar to what is observed for TEs – no correlation between piRNA biogenesis and expression level. However, an analogy may not be directly drawn here. TE transcripts are also involved in the generation of piRNA biogenesis, while host gene transcripts are not. The underlying mechanisms for the observed phenomena could be very different between TEs and host genes.

*When we went through our data to generate the revised analysis we discovered an error in our normalization code that explains the systematic loss of piRNAs associated with the D. simulans armi allele. As a result, we observe that genic piRNAs are less reduced with the simulans armi transgene when compared to D. melanogaster. Nevertheless, expanded our analysis of the relationship between off-target effects and piRNAs. We remain unable to uncover any apparent connection between the expanded negative regulation of protein coding genes associated with D. simulans armi, and changes in the abundance of antisense piRNA species for those same genes (Figure 4D of the revised manuscript). Thus, while simulans alleles appear to exhibit expanded negative regulation of host genes, this does not appear to result from increased targeting by piRNAs. Nevertheless we did uncover an increase in sense piRNAs derived candidate off targets of the D. simulans alleles, suggesting that off-target transcripts may be processed into piRNAs. In the revised manuscript we discuss these results in light of recent studies of off-target effects involving Piwi and Armi proteins, which arise through direct binding of the protein to host mRNA, and are apparently also independent of antisense piRNA.*

5. While I agree with the authors that their finding suggests a threshold effect of piRNA biogenesis (a very important finding!), it is still unclear why piRNA biogenesis may evolve adaptively. Horizontally transferred new TE families/escaped TE families may be one, as proposed by authors, but how the host machinery respond specifically to these families is still unclear. It would be helpful if the authors could further elaborate on this.

*We share the reviewer’s desire to understand the underlying cause of this evolutionary phenomenon. In the revised manuscript, we seek to provide a more thoughtful discussion of piRNA pathway evolution by contrasting our observations of interspecific divergence in cytoplasmic piRNA pathway proteins with recent analyses of divergence in the nuclear transcriptional silencers Rhino, Deadlock and Cutoff (Parhad et al. 2017, 2019). We emphasize the comparatively mild functional divergence among cytoplasmic factors as compared to their nuclear counterparts, as well as the evidence we uncover suggesting the potential importance of RNA-protein interactions in shaping the evolution of cytoplasmic factors. We further emphasize that our observations align with recent studies of piRNA protein evolution, which suggest that transcriptional silencing components evolve unusually rapidly among piRNA effector proteins (Palmer et a. 2018). Finally we echo the recent proposal that off-target effects play an underappreciated role in driving the evolution of both proteins transcriptional and cytoplasmic piRNA factors, but impose a stronger selective force on transcriptional regulators, due to their greater potentially to dramatically reduce the expression of host genes (Blumentiel et al 2016).*

Minor comments:

1. Figure 2C – where are these 92/43 TE families in Figure 1? Does this categorization correlate with piRNA abundance?

*These are TE families whose ping-pong fraction increases or decreases in the D. melanogaster transgenic rescue as compared to the armitage mutant. This is described in the figure legend and in the main text.*

2. I am a bit confused by Figure 2G, Aub panel. Why the two replicates show different trends (and they are both significant!)?

*Aubergine biological replicates were performed at different times separated by more than a year (which is mentioned in the Methods). We therefore presented the analysis of these libraries separately, since we observed some differences in size distribution that are likely related to differences in library prep. Nevertheless, the overall patterns are consistent across both replicates. For example in Figure 3G (formerly 2G) the +1U proportion is highest in the mutant, followed by the simulans transgenic rescue followed by the melanogaster transgenic rescue. This is consistent with a proportional decrease in ping-pong biogenesis, and corresponding proportional increase in phased piRNA biogenesis, in the simulans rescue and the mutant as compared to D. melanogaster.*

3. It would help the readers if authors could include a bit more descriptions of piRNA biogenesis through phasing mechanisms.

*Done, this discussion is now expanded in the Result section.*

4. 2x2 tables may be more informative than Figure 5A. (As the Y-axis is not labeled and only described in the figure legend).

*Done, these are now included in corresponding panel of the revised manuscript (figure 4B).*

5. Figure S2B does not provide enough support that the two transgenes have similar expression level – formal statistical tests are needed. It is also unclear which transgene was chosen as the baseline for calculating fold change (Y-axis).

*Statistical tests of differential expression are now provided. The only significant differential expression is observed with the SpnE transgene, where the D. simulans allele exhibits a modest (X-fold) increase in expression when compared to melanoagster. The D. melongaster transgene is the baseline.*

Reviewer #2: Summary

The authors aim to address the causes and consequences of positive selection on three piRNA pathway genes in Drosophila by creating transgenic rescue lines with D. melanogaster and simulans alleles. The manuscript is well-written and the experiment is an important one, and the results should be of great interest to those studying piRNAs in any species, irrespective of the outcome. Somewhat surprisingly, it appears rescuing D. melanogaster piRNA mutants with D. simulans alleles almost fully restores piRNA pathway function, with seemingly only slight differences in biogenesis and no differences in TE transcript abundance. The authors also find a difference in potential off-target effects – if true, a huge discovery, although more needs to be done to relate this to the other observed phenotypes, as well as confirmatory experiments that Dmel and Dsim proteins are at comparable levels.

Major comments

Overall, more needs to be done to better connect the observed phenotypes and create a clear picture of what the authors propose is happening. This should include both additional analysis (most of which should be able to be mined from available data) and writing, which are outlined separately below.

Analysis/Experiments:

1) It seems crucial that more should be done to confirm the experiment has worked as expected. For example, are there codon-bias or intron structure differences between melanogaster and simulans that could affect translation? While the authors show similar transcript levels, I think it is imperative that these Dsim and Dmel genes be shown to be translated at similar levels. Even small translation differences in theory could lead to the slightly impaired biogenesis phenotypes observed. Also, figure S2B could use positive controls.

*The reviewer is correct to point out that it is a common challenge with this sort of work to differentiate phenotypes that are associated with actual differences in protein structure vs. differential transcription and translation of encoded proteins. Western blots could potentially provide a semi-quantitative result. However they are subject to artifacts related to the differential cross-reaction of the antibody with the proteins of different species, which is a particular concern for adaptively evolving proteins such as the ones we investigate here. Nevertheless, at the request of this reviewer we looked into whether antibodies might be available to evaluate the relative protein abundance of transgenic rescues from both species. We have previously found that the Aub antibody* (*from Brennecke* et al*. 2007) does not cross react with the* D. simulans *allele*. *We obtained the available antibody for Armi (from Cook* et al. *2004), and similarly found that it does not cross react with the* D. simulans *allele. Spn-E antibodies are unfortunately not available.*

*We share the reviewer’s concern but would like to point out that we do not think that itundermines the major conclusion of our study, which is that the adaptive evolution in these three proteins has had surprisingly little impact on their functions with respect to piRNA production and TE regulation. If the modest defects in piRNA biogenesis and TE regulation that we observe are in fact due to differences in protein abundance, this would only make our conclusion more true. We also wish to point out the patterns we observe with D. simulans aub and armi alleles, where defects in piRNA maturation are associated with enhanced off target effects, are also not consistent with reduced protein abundance of D. simulans alleles. If protein abundance were reduced, piRNA maturation and off-target effects should both be reduced. We have added a brief discussion of this issue to the Discussion.*

2) The data presented supporting an autoimmune effect is relatively narrow in scope. Fleshing out this analysis to give a more global picture of what is happening to protein-coding genes during transgene rescue would help readers visualize exactly what is happening. For example, I am also interested in the number of genes that are upregulated by both transgene rescue – if similar numbers to those downregulated, then this may seem more like noisy RNA-Seq data or broader transcriptional dysregulation. If significantly more genes are downregulated upon rescue than upregulated, this would further support the autoimmunity hypothesis.

*Based on the comments of this reviewer as well as reviewer # we compared the # of genes that decreased in expression, as compared to increased in expression, in the D. melanogaster transgenic rescue as compared to the mutant . Consistent with negative regulation, significantly more protein coding genes were observed to decrease in expression as compared to increase in expression for all three proteins (Figure 4A). Specifically with respect to the concern about noisy RNA-seq data, given that these changes were consistent across 3 biological replicates for Armitage, and furthermore are largely consistent between two transgenic rescue alleles (mel and sim) sampling error doesn’t represent a plausible explanation for our observations.*

3) Second, it seems Figure 5 and Figure S6 should be combined into one analysis addressing the question: Does increased off-target effects lead to any meaningful differences in protein-coding gene expression? The authors show that the number of downregulated genes are more than expected (although I cannot find how “Expected” is calculated) and that the number of piRNA producing protein coding genes is more than expected in Dsim rescue, but these figures don’t seem meaningful if there is no overlap. I would also be interested to see how variable piRNA production from CDS is across replicates (for example, does gene X consistently produce more piRNAs upon Dsim rescue, or is a random draw of genes with higher piRNAs from each replicate).

*Expected counts were calculated under the assumption of statistical independence between two binomial variables transgene (mel or sim) and negative regulation (yes or no), as is typical for tests of independence. This is now stated in the legend of figure 4 in the revised manuscript.*

*Regarding the overlap between piRNAs and mRNAs, we think the reviewer may have misunderstood an important point. In fact in our original analysis there were fewer protein coding genes whose piRNA abundance increases in the simulans transgenic rescue (vs mutant) when compared to the melanogaster transgenic rescue (vs. mutant). This is the opposite of what you would predict if reduced protein coding gene expression results from increased piRNA production. In the revised manuscript, the difference in the number of protein coding genes whose piRNAs increase in the transgenic rescues is less pronounced after addressing the error in normalization that impacted the armitage data. Nevertheless it remains significant.*

*In the revised manuscript we more carefully interrogated the relationship between repressed transcription of protein coding genes and corresponding piRNA production on a gene by gene basis. We found no evidence that protein coding genes that were significantly negatively regulated by the sim transgene, but not the mel transgene, were more abundant among simulans antisense piRNAs (Figure 4D). As we discuss in our response to reviewer number 1 (point 4), this is fully consistent with recent studies of off-target effects, which suggest they occur in a piRNA-independent manner through the direct binding of piRNA proteins to target transcripts.*

4) Explanation: A couple of explanations could be provided to better connect the data, and frame it in what is already known or hypothesized. For example, the introduction (starting at P4L20) sets up competing hypotheses for what drives adaptation in piRNA pathway genes. It seems these options should be re-weighed in turn in the discussion. What scenarios is this data consistent with? Yes, various causes of positive selection but also e.g. differences in constraint between species, allowing divergence of neutral variation in Dsim that is functional in Dmel. Additionally, it is not clear how the authors believe genomic autoimmunity is occurring, and whether it is related to the decreased piRNA biogenesis. It would help readers to either explicitly say that these phenotypes have independent or correlated causes, and to integrate autoimmunity into the Figure 6 summary.

*We have considerably revised our discussion to more directly relate our observations to competing hypotheses about factors driving the evolution of piRNA pathway proteins.*

Minor comments

5) It seems discussing piRNA clusters and their protection would help general interest readers – without this knowledge the conundrum of why piRNA pathway genes are evolving adaptively is less apparent.

*It is our opinion that readers can understand that the specificity of piRNA silencing is conferred by piRNA species without needing to provide background information about piRNA clusters. We prefer to keep a more focused discussion that frames the research we performed.*

6) Why no ping-pong peak graphs?

*We prefer the box plots and heat maps that we present in figure 3 because they better capture variation among TE families with respect to ping-pong. This is particularly informative for analysis of the Armi, data, where ping-pong decreases or increases in transgenic rescues in a TE dependent manner.*

7) It would help general interest readers to include Spn-E in all of the figures where appropriate, and especially in the summary Figure 6.

*This point is moot since we have removed figure 6 from the revised manuscript, having revised our conclusions in light of the errors in armi piRNA analysis.*

8) On P9L19-20, a reference to Figure 2B should be included alongside 2F and G.

*Done.*

9) I had a very difficult time interpreting Figure 4B, perhaps explain it more thoroughly in the sentence starting on P13L18 “This counterintuitive positive…”, for example something along the lines of “notice how the colors relate in X way with the relationship between LFCs”

*This point is moot since we have removed the original figure 4 from the revised manuscript, having revised the focus of our work.*

10) It is not clear how expected numbers of autoimmune targets (Figure 5, S6) were calculated, this should be outlined. Also, is the observed significantly different from the expected? I only see a test done for comparing the two observed values, in which case, what are the expected values for?

*See our response to point 3, above.*

Discussion P20L9: Typo in melanogaster

*fixed.*

Reviewer #3: Please find the review document (Wang_et_al2019_review.docx) attached.

piRNA pathway proteins show signatures of adaptive evolution in Drosophila. What drives the rapid evolution of the piRNA machinery is unclear, but one prevailing hypothesis is that the host-TE arms race may contribute to the adaptive evolution of piRNA pathway factors. Functional consequences of rapid divergence have been studied for key piRNA factors though inter-species complementation tests, including an earlier study from the authors of this paper for Aub (Kelleher et al. 2012). Similar recent studies have tested the functional consequences of rapidly evolving nuclear piRNA factors Rhino, Deadlock (Parhad et al. 2017) and Cutoff (Parhad et al. 2019, bioarxiv) in inter-species complementation and structure-function tests to examine rescue of phenotypes with D. simulans alleles in D. melanogaster mutant background. This study is an interesting addition in testing functional consequences of rapid divergence in piRNA pathway components via inter-species complementation test, expanding on their earlier test (for Aub).

In this paper, Wang et al. test the functional consequences of rapid divergence of two additional cytoplasmic piRNA pathway factors armitage (armi) and spindle-E (spn-E). armi shuttles piRNA precursors between the ping-pong site (nuage) and the outer mitochondrial membrane where biogenesis of phased piRNAs occurs (Ge et al. 2019). Spn-E is helicase and co-IP experiments show they interact with other piRNA pathway factors such as Qin, Ago3 and Aub (Andress et al. 2016) indicating its role in piRNA amplification cycle in the nuage.

1. For fertility tests, the authors show D. simulans alleles are sub-fertile (sterility is rescued), but it seems that in terms of phenotype rescue, the adaptive evolution in the cytoplasmic factors (tested here) are different from nuclear factors like Rhino, Deadlock and cutoff, where D. simulans alleles fail to rescue sterility phenotype. I am raising this to know if these piRNA factors were to be ranked based on dN/dS or other types of selection tests the authors performed, do nuclear factors experience more positive selection than the cytoplasmic ones?

*There is a recent meta-analysis (Palmer* et al*. 2018) which does suggest that transcriptional silencing factors evolve more rapidly than the biogenesis factors (Armi and Spn-E) and post-transcriptional effectors (Aub) that we examined here. Particularly in light of our revised observation that none of the three proteins we examined exhibit dramatic defects in piRNA production or TE regulation, as compared to previously examined transcriptional regulators (Rhino, Deadlock, Cutoff), in our revised discussion we speculate about how and why adaptive evolution may differ between these two groups of proteins,*

I see Rhino-deadlock work mentioned in the discussion leading to figure 6, but also work on another interacting factor cutoff is now on Bioarxiv that the authors can include in the discussion. It would be informative to include details as a rationale for why the authors chose these factors for complementation tests. Do they have significant changes in their critical domains? This information would be very helpful to interpret functional consequences, as recent co-IP studies (Andress et al. 2016, Ge et al. 2019) show that these factors interact with a host of other piRNA biogenesis and non-piRNA pathway proteins.

*We thank the reviewer for bringing this pre-print to our attention and have cited it multiple times in our revised manuscript. We also attempted to more clearly lay out the functional distinction between the SpnE, Aub, Armi and Rhino Cutoff and deadlock, as well as more sharply distinguish their patterns of evolution and functional divergence. We also speculate as to possible factors driving these differences in our revised discussion.*

*With regards to changes in functional domains, amino acid substitutions between the alleles we examined are observed throughout the sequence of each protein, both inside and outside of funcitonal domains. This is now represented in figure 1A of the revised manuscript.*

The authors propose a model for armi suggesting its enhanced affinity to binding ago3/aub may explain the phenotype observed (reduced ping-pong, and reduction in phased piRNAs), but it seems from co-IP that spn-E also interacts with armi, ago3 and aub, and perhaps co-evolution to maintain a robust interaction complex in the cytoplasm an additional target of selection? In the absence of structure-function tests for these piRNA factors, a detailed picture of where non-synonymous changes lie (e.g. concentrated on critical domains, or spread across the entire gene) within these genes, and whether they impact interactions with other genes can be included and discussed.

*This is now provided in figure 1A.*

2. For Figure 1, please include the number of TE families (circles) in the panel and the “n” that is changing. At 2-fold difference how many TE families exhibit a change in the armi D. simulans rescue? I noticed the top panel is 2-fold difference and the bottom one is 1.5, is there a reason for having different fold difference cutoff? If not, just use 1.5 fold difference. Also, it would be very helpful to evaluate a supplementary table with library mapping statistics for the small RNA data. In addition, as the authors observe a drop in piRNA pool in the D. simulans armi rescue, it would be helpful to see small RNA mappings to different annotations as a pie-chart (miRNAs, coding, etc.) so I can evaluate what fraction of piRNA pool is lost in these libraries (and eventually taken-up by other categories in the library). I agree with miRNA normalization for these datasets, but it would be helpful to see the mapping fraction of libraries to different annotations for all datasets as a supplementary figure.

*At the suggestion of this reviewer we changed our log2FC cutoff to be 2 for all comparisons, since we found that that better emphasized the difference between mutants and transgenic rescues, and focused our discussion of differentially expressed TEs to the most extreme cases. We included the numbers of differentially expressed TEs in the revised manuscript (Figure 2).*

3. For figure2, was the experiment designed to evaluate ping-pong and phasing in WT, so the authors can add a comparison of mutant, WT, mel-rescue and sim-rescue? I agree the authors were careful in integrating transgenes into matched attP sites, but if the data is the available, it would be helpful to evaluate any background effects in ping-pong and phasing between WT and mel-rescue.

*See our response to reviewer number 1, comments 1 and 2. The revised manuscript includes a comparisons of our transgenic rescues to heterozygotes, which are commonly considered wild-type in genetic analysis of piRNA pathway protein function (Figure S3).*

4. Same as figure1, for figure 3, please include “n” changing in the figure panel, and use consistent 1.5 or 2 fold differences.

*Done, these are now provided in the corresponding figure of the revised manuscript (Figure 2).*

5. For the off-targets effects, it was hard for me to evaluate this result without the details from differential expression analysis. It would be helpful to evaluate if the authors provided a supplementary table with the genes that show up and down regulation. I am not entirely convinced by this result, perhaps due to lack of underlying data to evaluate the results. The authors do not observe any up regulation of genes with D. simulans and/or D. melanogaster rescue at all? For any RNA-seq analysis, I am assuming there will be indirect effects on genes that are up/down regulated, so a comparison of D. melanogaster WT and D. melanogaster rescue RNA-seq would be a good control to assess background effects, if any. Also, for the “candidates” for off-target effects, please provide examples as a supplementary figure to show complementarily, number of piRNAs detected etc. A related question is that did the authors enrich 3’ modified small RNAs for small RNA sequencing. If not, the authors can discuss based on mapping to these protein-coding genes that they are not likely degradation of mRNAs, but genuine piRNAs. Also it is not clear from figure 4 if all the down-regulated genes are targeted by piRNAs, or some of these just secondary effects in down-regulation of genes?

*The revised manuscript includes a new panel (Figure 4A) which reveals the proportion of genes that are upregulated or downregulated in transgenic rescues as compared to mutants. We furthermore carefully interrogated the relationship between off-target effects and piRNAs (see our response to reviewer #2, comment 3, reviewer #1 comment 4) and did not observe a relationship between off target effects and piRNA production. As stated more thoroughly above, this is fully consistent with recent analysis of off-target effects of cytoplasmic piRNA proteins, as we discuss extensively in the revised manuscript.*

Minor comments:

Figure 5A, label Y-axis (no. of genes).

*Done.*

Figure S6, label Y-axis (no. of genes).

*Done.*

Supplementary data tables for the underlying small RNA and RNA-seq data, both library stats and processed downstream files would be very helpful to evaluate results.

Figure 3 and figure S5, the authors acknowledge idiosyncratic differences in expression of individual TE families and conclude majority of TEs are not differentially expressed. I agree with this result, but the yellow (dots in figure 3, and bars in figure S5) are from D. melanogaster rescue. It would be very useful to make a comparison between WT and D. melanogaster rescue so just background effects can be evaluated. This is an important control in assessing background effects, and reflects my comments made in point 3.

The authors can cite Parhad et al. (Bioarxiv; complementation test for cutoff) in the discussion.

*We have cited this important emerging work at multiple places in the revised manuscript.*

*We aren’t sure what the reviewer means by background effects, since by backcrossing the melanogaster and simulans transgenes into the same mutant stocks we have very carefully controlled for background differences among the genotypes we compare. Therefore, although particular TE families could be more abundant in the backgrounds we chose, these would not contribute to differences among genotypes.*

Reviewer #4: Overall, the conclusions of this paper are well supported. However, it would be worth outlining some caveats to these interpretations. In addition, in parts, the presentation of the data could be more clear. In places, the impact of the results is somewhat hidden in the visuals.

1. Could the authors provide an alignment of the protein sequences of the sim and mel alleles? The authors may want to relegate this to the supplemental, but unless these alignments are unwieldy (hopefully they can be presented in a way they are not), I would recommend they put these alignments in the first figure. Without this, the reader is left wondering what kind of divergence there is between the sim and mel protein sequences.

*In the revised manuscript we provide a new figure relating functional domains to amino acid differences between the alleles we compared (Figure 1). We have also increased our discussion of previously published analysis of positive selection acting on the coding sequences of these genes (Obbard* et al. *2009; Kolaczkowski* et al*. 2011; Simkin* et al. *2013).*

2. One of the main results is that the sim-armi allele leads to a universal reduction of piRNA abundance against TEs. Since this is normalized against total sequence reads, what small RNA class is increased in their place? In the methods, it is stated that TE-piRNA counts were normalized to miRNA. It would be worth reminding the reader that this class was increased as a proportion of total small RNA reads.

*This point is somewhat moot since the universal reduction in TE derived piRNAs was a computational artifact that has been addressed in the revised manuscript. Nevertheless we did include size histograms of piRNA libraries in Figure S4 of the revised manuscript.*

3. I feel the scatter plot buries the observation of reduced piRNA amounts. Can a miRNA-normalized size histogram of piRNAs mapping to TEs be provided for each of the alleles? That way, the reader can see the whole distribution presumably downshifted for the sim variants - especially for armi. This would hide some of the TE level variation for sim spnE, but would be a helpful view of total piRNA amounts mapping to TEs. Overall, for TE mapping piRNAs, the reader needs to also have a fold reduction number (50% reduction or whatever) for each of the alleles that they can grasp. Especially since it is against this fold reduction of piRNA that the TE expression will be measured against.

*Please see above reply.*

3. Leading into the TE expression section, the authors should remind the reader about the total fold reduction in piRNA abundance for the three sim alleles and ask if there is a proportional increase in TE expression. Again, it would be helpful to get a single metric for fold change in TE expression across the TE bulk (perhaps for just the TE that are misregulated in the mutants stocks). The point I think the authors are trying to make is that a proportional decrease in total piRNA amounts doesn't lead to a proportional increase in TE expression. To make that argument, the figures as presented are not sufficient. They need to present these numbers directly in the text for bulk decrease of TE piRNAs and bulk increase for TE expression.

*This point is moot for the same reason as above two points. Nevertheless we restructured the manuscript such that the TE regulation and piRNA abundance data are presented in a combined figure (Figure 2), and clarify our dicussion of the relationship between these two pools of transcripts.*

4. For figure 4B, Dmel-armi vs. Dsim-armi, the scale for log2FC TE transcript abundance should be on the same level as in 4A for spnE and aub. As presented, it gives the impression that the variance for log2FC TE expression is higher for armi. Maybe scale all of them from -1 to 2?

*This point is moot since we have removed the original figure 4 from the revised manuscript, having revised the focus of our work.*

5. Based on Figure 4A, in the text the authors state (page 13, line 13): Strikingly, we observed no correlated changes between piRNA and mRNA pools for aub and spnE when the two transgenic rescues are compared to each other, suggesting the magnitude of changes in piRNA abundance are not sufficient to impact downstream targets.

I feel like the use of the word "Strikingly" is misleading. If one looks at the range of piRNA level change in the mutant vs. transgene that is equivalent in the transgene vs. transgene range, one also doesn't see a relationship in the mutant vs. transgene plots. So, clearly the magnitude of piRNA abundance change is not sufficient in the transgene vs. transgene contrast to reveal this relationship. The way the figure is set up, it might imply to the reader that there is a difference in the relationship between piRNA abundance and expression. But, clearly the results do support the conclusion that TE control is robust to changes in piRNA abundance in this range.

*We take the reviewers point, however, we have revised this section considerably in light of the computational artifact that impacted our armi piRNA analysis. Figure 4 of the original manuscript is no longer included in the revised manuscript.*

6. Why does 4A examine Log2FC for total TE transcript abundance by 4B look at antisense transcript abundance?

*See above*

7. Regarding genic piRNAs - perhaps I don't follow Figure 6B, but in a direct contrast of sim vs. mel alleles, are there more genic piRNAs in one vs. the other? 6B seems to be relative to mutant, but what about vs. each other? Perhaps some clarification of this would be helpful.

*In the revised manuscript we compare the abundance of genic antisense piRNAs corresponding to host genes that are repressed by D. simulans alleles but not by D. mel alleles (Figure 4D). For aub and spnE. we did not observe that these log2FC values differed significantly from zero, indicating that there is no systematic change in piRNAs associated with off-target effects. For armi, we observed that antisense genic piRNAs were more abundant in the presence of the D. melanogaster transgene, which is not consistent with off-target effects resulting from upstream changes in antisense piRNA.*
