# Supplementary material for: Adaptive evolution among cytoplasmic piRNA proteins leads to decreased genomic auto-immunity
Source: PLoS Genet. 2020 Jun 11;16(6):e1008861. doi: 10.1371/journal.pgen.1008861 (PMC7310878; doi:10.1371/journal.pgen.1008861)
Supplement: Attachment — Submitted filename: Wang_et_al_revision_review.docx [file pgen.1008861.s021.docx]

1. For fertility tests, the authors show D. simulans alleles are sub-fertile (sterility is rescued), but it seems that in terms of phenotype rescue, the adaptive evolution in the cytoplasmic factors (tested here) are different from nuclear factors like Rhino, Deadlock and cutoff, where D. simulans alleles fail to rescue sterility phenotype. I am raising this to know if these piRNA factors were to be ranked based on dN/dS or other types of selection tests the authors performed, do nuclear factors experience more positive selection than the cytoplasmic ones?

*There is a recent meta-analysis (Palmer* et al*. 2018) which does suggest that transcriptional silencing factors evolve more rapidly than the biogenesis factors (Armi and Spn-E) and post-transcriptional effectors (Aub) that we examined here. Particularly in light of our revised observation that none of the three proteins we examined exhibit dramatic defects in piRNA production or TE regulation, as compared to previously examined transcriptional regulators (Rhino, Deadlock, Cutoff), in our revised discussion we speculate about how and why adaptive evolution may differ between these two groups of proteins*

The expanded discussion in the revised manuscript now addresses this concern.

I see Rhino-deadlock work mentioned in the discussion leading to figure 6, but also work on another interacting factor cutoff is now on Bioarxiv that the authors can include in the discussion. It would be informative to include details as a rationale for why the authors chose these factors for complementation tests. Do they have significant changes in their critical domains? This information would be very helpful to interpret functional consequences, as recent co-IP studies (Andress et al. 2016, Ge et al. 2019) show that these factors interact with a host of other piRNA biogenesis and non-piRNA pathway proteins.

*We thank the reviewer for bringing this pre-print to our attention and have cited it multiple times in our revised manuscript. We also attempted to more clearly lay out the functional distinction between the SpnE, Aub, Armi and Rhino Cutoff and deadlock, as well as more sharply distinguish their patterns of evolution and functional divergence. We also speculate as to possible factors driving these differences in our revised discussion. With regards to changes in functional domains, amino acid substitutions between the alleles we examined are observed throughout the sequence of each protein, both inside and outside of funcitonal domains. This is now represented in figure 1A of the revised manuscript.*

The authors propose a model for armi suggesting its enhanced affinity to binding ago3/aub may explain the phenotype observed (reduced ping-pong, and reduction in phased piRNAs), but it seems from co-IP that spn-E also interacts with armi, ago3 and aub, and perhaps co-evolution to maintain a robust interaction complex in the cytoplasm an additional target of selection? In the absence of structure-function tests for these piRNA factors, a detailed picture of where non-synonymous changes lie (e.g. concentrated on critical domains, or spread across the entire gene) within these genes, and whether they impact interactions with other genes can be included and discussed.

*This is now provided in figure 1A*

This concern is now addressed in Figure 1A and the discussion in the revised manuscript.

2. For Figure 1, please include the number of TE families (circles) in the panel and the “n” that is changing. At 2-fold difference how many TE families exhibit a change in the armi D. simulans rescue? I noticed the top panel is 2-fold difference and the bottom one is 1.5, is there a reason for having different fold difference cutoff? If not, just use 1.5 fold difference. Also, it would be very helpful to evaluate a supplementary table with library mapping statistics for the small RNA data. In addition, as the authors observe a drop in piRNA pool in the D. simulans armi rescue, it would be helpful to see small RNA mappings to different annotations as a pie-chart (miRNAs, coding, etc.) so I can evaluate what fraction of piRNA pool is lost in these libraries (and eventually taken-up by other categories in the library). I agree with miRNA normalization for these datasets, but it would be helpful to see the mapping fraction of libraries to

different annotations for all datasets as a supplementary figure.

*At the suggestion of this reviewer we changed our log2FC cutoff to be 2 for all comparisons, since we found that that better emphasized the difference between mutants and transgenic rescues, and focused our discussion of differentially expressed TEs to the most extreme cases. We included the numbers of differentially expressed TEs in the revised manuscript (Figure 2).*

This concern is now addressed in Figure 2 and Figure S5

3. For figure2, was the experiment designed to evaluate ping-pong and phasing in WT, so the authors can add a comparison of mutant, WT, mel-rescue and sim-rescue? I agree the authors were careful in integrating transgenes into matched attP sites, but if the data is the available, it would be helpful to evaluate any background effects in pingpong and phasing between WT and mel-rescue.

*See our response to reviewer number 1, comments 1 and 2. The revised manuscript includes a comparisons of our transgenic rescues to heterozygotes, which are commonly considered wild-type in genetic analysis of piRNA pathway protein function (Figure S3).*

This concern is addressed in Figure S3. As the authors mentioned, piRNA effector proteins are haplosufficient and hets can be used as a WT control. In figure S3A it is not clear from the legend whether the plot is for piRNA abundance in the library or piRNA abundance to TE transcripts (similar to figure 2B). Also, can the authors include a panel in figure S3 similar to figure 2B just comparing piRNA abundance between Dmel-rescue and hets with a 2-fold line.

4. Same as figure1, for figure 3, please include “n” changing in the figure panel, and use consistent 1.5 or 2 fold differences.

*Done, these are now provided in the corresponding figure of the revised manuscript (Figure 2).*

This is now addressed in the revised manuscript

5. For the off-targets effects, it was hard for me to evaluate this result without the details from differential expression analysis. It would be helpful to evaluate if the authors provided a supplementary table with the genes that show up and down regulation. I am not entirely convinced by this result, perhaps due to lack of underlying data to evaluate the results. The authors do not observe any up regulation of genes with D. simulans and/or D. melanogaster rescue at all? For any RNA-seq analysis, I am assuming there will be indirect effects on genes that are up/down regulated, so a comparison of D. melanogaster WT and D. melanogaster rescue RNA-seq would be a good control to assess background effects, if any. Also, for the “candidates” for off-target effects, please provide examples as a supplementary figure to show complementarily, number of piRNAs detected etc. A related question is that did the authors enrich 3’ modified small RNAs for small RNA sequencing. If not, the authors can discuss based on mapping to these protein-coding genes that they are not likely degradation of mRNAs, but genuine piRNAs. Also it is not clear from figure 4 if all the down-regulated genes are targeted by piRNAs, or some of these just secondary effects in down-regulation of genes?

*The revised manuscript includes a new panel (Figure 4A) which reveals the proportion of genes that are upregulated or downregulated in transgenic rescues as compared to mutants. We furthermore carefully interrogated the relationship between off-target effects and piRNAs (see our response to reviewer #2, comment 3, reviewer #1 comment 4) and did not observe a relationship between off target effects and piRNA production. As stated more thoroughly above, this is fully consistent with recent analysis of off-target effects of cytoplasmic piRNA proteins, as we discuss extensively in the revised manuscript.*

Similar to small RNA data comparisons for het vs. mel-rescue (Malone et al. 2009), RNA-seq datasets for het or WT vs mutant (aub, armi and spn-E) are available. As a control, it would be helpful to show a comparison between het vs mel-rescue for RNA-seq data. Or, if the authors can show from previous studies comparing mut vs. het (aub, armi and spn-E) that the overlap in genes up/downregulated is the nearly the same as mel-rescue vs. mutant comparison (and that more genes tend to be downregulated than upregulated), this will be additional support that mel-rescue indeed behaves as het (WT) for RNA-seq data.

In the revised manuscript, the authors observe off-target effects are not due to piRNAs (lack of evidence for anti-sense piRNAs to mRNAs), but instead observed an increase in sense piRNAs, which is consistent with a model of promiscuous binding of piRNA pathway proteins to process mRNAs into piRNAs. Figs. 4 D and E are not very informative. Are the blue and grey dotted lines expected vs. observed? This is not mentioned in the figure legend. Wouldn’t it be more informative if the authors show this result similar to Fig. 2B? This would be a nice locus-by-locus comparison of sense piRNA levels like the authors do for TE anti-sense piRNAs in Fig. 2B.

Also, in table S9 if the log2FC values are interpreted as 2-fold changes for sense piRNA production (similar to what the authors do for TEs), what fraction of genes in aub and armi have 2-fold more sense piRNAs in D.sim rescue compared to D.mel rescue? If the comparisons are from DEseq2, provide adjusted P for every gene where replicates are available, similar to table S3. If promiscuous binding of piRNA effector proteins generates more sense piRNAs from mRNAs on a model that the authors propose, wouldn’t there be significantly more sense piRNAs (1.5 fold or more) for every individual downregulated locus in D.sim rescue compared to D.mel rescue (similar to anti-sense piRNAs for TEs; fig 2B)? From table S9, I am not sure if the data strongly support this model.

One other possibility is that binding of piRNA effector proteins to mRNAs may promote mRNA degradation (see Barckmann et al. 2015). As the suggested selection for genomic autoimmunity is a critical point in this manuscript, the authors can examine if the downregulated genes in their comparison are bound by aub in the aub iCLIP data in Dmel from Barckmann et al. 2015 to support this model (the authors show large fraction of downregulated genes are shared between Dmel and Dsim; Fig 4C). The data is from 0-2hr embryos, but mRNA population in the ovary is represented in the maternally deposited pool, and either this or analysis of similar CLIP data may lend strong support for the model that the authors propose in this manuscript.

Minor comments:

Figure 5A, label Y-axis (no. of genes).

*Done.*

Figure S6, label Y-axis (no. of genes).

*Done.*

Supplementary data tables for the underlying small RNA and RNA-seq data, both library stats and processed downstream files would be very helpful to evaluate results. Figure 3 and figure S5, the authors acknowledge idiosyncratic differences in expression of individual TE families and conclude majority of TEs are not differentially expressed. I agree with this result, but the yellow (dots in figure 3, and bars in figure S5) are from D. melanogaster rescue. It would be very useful to make a comparison between WT and D. melanogaster rescue so just background effects can be evaluated. This is an important control in assessing background effects, and reflects my comments made in point 3.

The authors provide supplementary data tables for sRNA and RNA-seq data in the revised manuscript. For table S9, if the comparisons are from DEseq2, provide adjusted P similar to table S3 and S8.

The authors can cite Parhad et al. (Bioarxiv; complementation test for cutoff) in the discussion.

*We have cited this important emerging work at multiple places in the revised manuscript.*

*We aren’t sure what the reviewer means by background effects, since by backcrossing the melanogaster and simulans transgenes into the same mutant stocks we have very carefully controlled for background differences among the genotypes we compare. Therefore, although particular TE families could be more abundant in the backgrounds we chose, these would not contribute to differences among genotypes.*
